# Exploring patterns of recurrent melanoma in Northeast Scotland to inform the introduction a digital self-examination intervention

**DOI:** 10.1186/1471-5945-14-4

**Published:** 2014-03-11

**Authors:** Rhona Auckland, Patrick Wassell, Susan Hall, Marianne C Nicolson, Peter Murchie

**Affiliations:** 1 School of Biomedical Sciences, Medical School, Teviot Place, Edinburgh EH8 9AG, UK; 2 Medical School, University of Aberdeen, Polwarth Building, Foresterhill, Aberdeen AB25 2ZD, UK; 3 Centre of Academic Primary Care – Division of Applied Health Sciences, University of Aberdeen, Polwarth Building, Foresterhill, Aberdeen AB25 2ZD, UK; 4 ANCHOR Unit, Aberdeen Royal Infirmary, Foresterhill, Aberdeen AB25 2ZN, UK

**Keywords:** Melanoma recurrence, Self-detected, Follow-up, Skin self-examination, Education

## Abstract

**Background:**

Melanoma incidence is growing and more people require follow-up to detect recurrent melanoma quickly. Those detecting their own recurrent melanoma appear to have the best prognosis, so total skin self examination (TSSE) is advocated, but practice is suboptimal. A digital intervention to support TSSE has potential but it is not clear which patient groups could benefit most. The aim of this study was to explore cutaneous melanoma recurrence patterns between 1991 and 2012 in Northeast Scotland. The objectives were to: determine how recurrent melanomas were detected during the period; explore factors potentially predictive of mode of recurrence detection; identify groups least likely to detect their own recurrent melanoma and with most potential to benefit from digital TSSE support.

**Methods:**

Pathology records were used to identify those with a potential recurrent melanoma of any type (local, regional and distant). Following screening of potential cases available secondary care-held records were subsequently scrutinised. Data was collected on demographics and clinical characteristics of the initial and recurrent melanoma. Data were handled in Microsoft Excel and transported into SPSS 20.0 for statistical analysis. Factors predicting detection at interval or scheduled follow-up were explored using univariate techniques, with potentially influential factors combined in a multivariate binary logistic model to adjust for confounding.

**Results:**

149 potential recurrences were identified from the pathology database held at Aberdeen Royal Infirmary. Reliable data could be obtained on 94 cases of recurrent melanoma of all types. 30 recurrences (31.9%) were found by doctors at follow-up, and 64 (68.1%) in the interval between visits, usually by the patient themselves. Melanoma recurrences of all types occurring within one-year were significantly more likely to be found at follow-up visits, and this remained so following adjustment for other factors that could be used to target digital TSSE support.

**Conclusions:**

A digital intervention should be offered to all newly diagnosed patients. This group could benefit most from optimal TSSE practice.

## Background

Melanoma incidence has risen over the last 50 years, and disproportionately affects younger people [1]. Around three times as many cases were reported in 2000 than in 1970 and it is now the sixth commonest cancer in the UK [2,3]. Scottish guidelines recommend that people treated for cutaneous melanoma receive stuctured follow-up consisting of regular physical examination by a specialist without blood tests or imaging unless subsequently indicated [4]. Follow-up aims to detect melanoma recurrences early and expedite secondary care access if necessary and its delivery is becoming increasingly burdensome to healthcare systems [5-7]. Many recurrences are detected in the interval between structured follow-up visits leading many to question it value [8,9]. On the other hand there is evidence that most early recurrences (within two years) are not self-detected but found at scheduled follow-up appointments [8,10-13]. This is important since there is evidence of superior survival rates of those who self-detected their recurrence appear to have superior survival, corresponding with with findings that regular total skin self examinations (TSSE) in people treated for primary cutaneous melanoma can reduce mortality rates by as much as 63% [13,14].

Despite this TSSE education and practice appear suboptimal with 70% of American melanoma patients indicating that they had never been advised to do it [15]. There is good reason to suggest that similar statistics would be found in North East Scotland (NES). Where interventions to improve TSSE have been tried, results have been disappointing and those who were educated by brochure or video demonstrations only reported increased TSSE practice for 3–7 months, with overall participation returning to the baseline by 12 months [16-18]. Despite this it is encouraging that educational interventions could achieve 17-50% increases in TSSE practice, albeit in the short-term. Further, stabilisation of mortality in younger patients (aged 25–44), despite increasing incidence, is thought to result from increased public awareness and TSSE promotion [1,16,19,20]. Similarly higher survival and higher TSSE rates are observed in less deprived people [15]. All this supports the view that TSSE is worth doing. Some suggest that digital interventions could promote and sustain TSSE practice [18-20]. Such interventions however, are likely to be expensive to develop and implement so should be targeted at those with the greatest potential to benefit, information which the current studies do not provide.

We are developing a digital intervention to promote and prompt TSSE in Northeast Scotland. We wished to explore patterns of all types of melanoma recurrence within the region over recent years to determine when, and to which patients, this intervention should be targeted.

## Methods

### Study approval

Formal approval for this study was granted on 10^th^ October 2012 by the Quality, Governance and Risk Unit (Clinical Effectiveness Team) of NHS Grampian (project ID 2483).

### Identifying recurrences

A data-base maintained by the Department of Pathology, NHS Grampian was scrutinised to identify melanoma patients from Northeast Scotland potentially diagnosed with any type of recurrence between August 1992 and September 2012.

### Data collection

A data collection sheet was constructed (Table 1) and used to abstract data from the secondary care-held medical records of eligible and available cases at the medical records department at Aberdeen Royal Infirmary.

**Table 1:** Melanoma recurrence data sheet

Surname:	DOB:	
Forename(s):	Gender:	
CHI:		
Occupation:	Ethnicity:	
Date of primary melanoma diagnosis:		
Melanoma type:		
Stage of melanoma:		
Tumour thickness (mm):	Breslow depth;	
	Clark level;	
Prognostic features:	Ulceration;	
	Lymph node involvement;	
	Tumour vascularity;	
	lymphovascular invasion;	
	Mitotic rate;	
	Regression;	
	Microsatellites;	
	Tumour-infiltrating lymphocytes;	
	Lactate dehydrogenase serum level;	
Tumour anatomical location:		
Method/Details of treatment:		
Details on how the melanoma was picked up:		
Date of excision biopsies:	Primary biopsy;	
	Secondary biopsy;	
	Punch/Shave biopsy;	
Details of prescribed follow-up program:		
Date of recurrence diagnosis:		
Nature of recurrence: local, regional, distant)		
Melanoma type:		
Melanoma stage:		
Tumour thickness:	Breslow depth;	
	Clark level;	
Prognostic features:		
Tumour anatomical location:		
How was the recurrence detected:		Please Tick
	Self – detected	
	GP	
	Dermatologist	
	Follow-up appointment	
	Other	
If other, please state:		
If self- detected, which of the following apply:		Please Tick:
Recurrence found via;	Routine self-examination	
	Accidental find	
	Aid of partner/Acquaintance	
	Experience of related symptoms (e.g. nausea, fatigue, weight loss, pain, shortness of breath etc.)	
Route from self-detection to official diagnosis: (e.g. Patient – GP – Hospital)		
Dates of above referrals: (e.g. GP referral, treatment dates etc.)		

The following data were abstracted (Table 2):

i) Demographics: gender; date of birth; age at diagnosis; age at recurrence; postcode. Postcode was subsequently use to define deprivation and rurality [21,22].

ii) Clinical details of the initial melanoma including: melanoma type; Breslow thickness; anatomical location.

iii) Type of recurrence: local; regional; distant.

iv) Recurrence pathway: time to recurrence; mode of detection (self detected and reported during interval; found at follow-up clinic by clinician; emergency admission during interval; other).

**Table 2:** Clinicopathological Characteristics; descriptive statistics in relation to frequency within sample of 94 patients

	**Frequency:**
**Demographics:**	
***Gender;***	
Female	37 (39.4%)
Male	57 (60.6%)
***Deprivation score;***	
1(most deprived)	5 (5.3%)
2	9 (9.6%)
3	22 (23.4%)
4	27 (28.7%)
5(least deprived)	31 (33.0%)
***Six-fold rurality score;***	
Urban	43 (45.6%)
Rural	51 (54.4%)
***Type of recurrence***	
Local	21 (22.3%)
Regional	48 (51.1%)
Distant	25 (26.6%)
**Details of first primary:**	
***Age at diagnosis;***	
<=50 years	19 (20.2%)
51-70 years	41 (43.6%)
71+ years	33 (35.1%)
Unknown	1 (1.1%)
***Melanoma type of first primary;***	
Superficial spreading	27 (28.7%)
Nodular malignant	30 (31.9%)
Lentigo maligna	5 (5.3%)
Acral lentiginous	5 (5.3%)
Other	27 (28.7%)
***Location of first primary;***	
Lower limbs	35 (37.2%)
Trunk	20 (21.3%)
Upper limbs	9 (9.6%)
Head and neck	22 (23.4%)
Mucus membranes	4 (4.3%)
Eye	4 (4.3%)
***Breslow thickness of first primary;***	
<0.75 mm	1 (1.1%)
0.76-4.00 mm	18 (19.1%)
>4.00 mm	36 (38.3%)
Unknown	27 (28.7%)
	12 (12.8%)
**Details of recurrence:**	
***Age at recurrence;***	
<=50 years	14 (14.9%)
51-70 years	40 (42.6%)
71+ years	40 (42.6%)
***Time to recurrence;***	
0-12 months	27 (28.7%)
13-24 months	28 (29.8%)
25-36	11 (11.7%)
37-48	5 (5.3%)
49-60	7 (7.4%)
5+ years	15 (16.0%)
Unknown	1 (1.1%)
***How recurrence was detected;***	
Self-detected and reported during interval	45 (48.9%)
New finding at follow-up clinical	30 (31.9%)
Emergency admission with symptoms in interval	11 (11.7%)
Other	5 (5.3%)

Data were entered handled using a Microsoft Excel worksheet. After scrutiny for errors they were read into SPSS version 20 for further statistical analysis.

### Statistical analysis

#### Demographic and clinical characteristics

Basic descriptive statistics of the sample were prepared. Demographics characteristics, clinical characteristics of the primary melanoma and details of the recurrence and its detection pathway were explored.

#### Categorising variables

To conduct meaningful univariate analysis on this relatively small sample was challenging. A number of categorical variables were created from the data to address this. After discussion amongst the authors a binary outcome variable was created distinguishing those patients who had their recurrent melanoma detected de-novo at the follow-up clinic versus those in whom the presentation of recurrence had occurred in the interval between scheduled follow-up visits. Arguably, the former group are those who stand most to gain from interventions to promote total skin self examination.

Seven predictor variables were then created. These were selected on the basis that there was evidence for, or it seemed plausible that, they could be influential on whether recurrent melanoma was detected at scheduled follow-up or during the interval between scheduled follow-up appointments. Candidates were classified by gender, 5-fold deprivation score and 2-fold rurality score. With reference to recurrent melanoma, variables including age (<65 versus ≥65 years) time to recurrence (< 1 year versus ≥ 1 year) were considered and details of the initial melanoma (subtype, Breslow thickness (<4 mm; ≥4 mm)) [8].

#### Univariate analysis

The outcome variable (detected at scheduled follow-up or not) was cross-tabulated with each of the six predictor variables. The chi-squared test was used to ascertain if the six factors were significantly associated with follow-up versus interval detection, with statistical significance being designated as a p ≤ 0.05. The strength of the relationship between the outcome and each of the six predictor variables was also explored univariately using binary logistic regression to generate odds ratios and 95% CIs.

#### Multivariate analysis

A multivariate binary logistic regression model was then constructed including all factors found to be significantly predictive of follow-up versus interval recurrence detection, as well as those factors that could conceivably inform targeting of a digital intervention to support TSSE (Breslow thickness; time to recurrence; age at recurrence; rurality; deprivation score).

## Results

### Included and excluded cases

**Figure 1:**
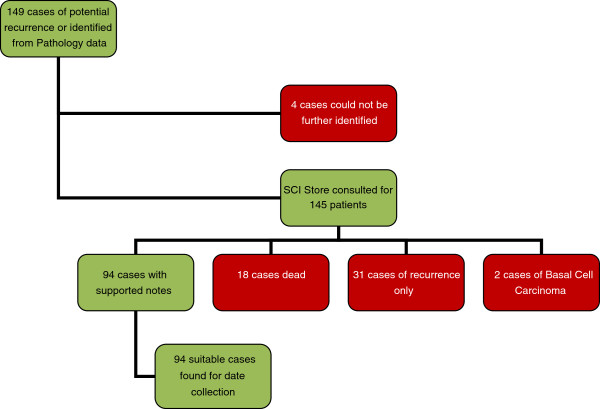
Study flow.

149 potential cases of any type of recurrent melanoma were identified (Figure 1). Eighteen people had died and notes were not available and four sets of notes could not be located. Thirty-one people had recurrent melanoma with no record of a prior primary. Two further apparent recurrences proved to be new basal cell carcinomas on review of pathology data. This resulted in 55 exclusions and a final sample of 94 cases for analysis.

### Demographic and clinical characteristics

Demographics and characteristics of primary melanomas are shown in Table 2. The sample included 21 (22.3%) local, 48 (51.1%) regional and 25 (26.6%) distant recurrences. Mean age at the time of recurrence was 65 years. Forty-five recurrences (58.5%) had presented within two years of diagnosis. Thirty (31.9%) cases were detected at scheduled melanoma follow-up appointments. The remaining 64 (69.1%) were detected as interval events with 45 (48.9%) being obviously self detected, 11 (11.7%) being emergency admissions to hospital with metastatic disease and five (5.3%) being detected through another route, mostly incidental findings.

### Univariate analysis

Those variables deemed most likely to affect whether a recurrence was found at follow-up or self detected in the interval were explored univariately with the chi-squared test (Table 3). The most striking finding was earlier recurrences were significantly more to be detected at structured follow-up. Of recurrences within a year of diagnosis 51.9% were detected at follow-up, while only 23.9% of later recurrences were detected at follow-up. No other potential predictors of recurrence detection location were significant univariately.

**Table 3:** Cross tab data analysis; determining the significance of categorised variables on likelihood to present at follow-up or in interval

**Descriptive statistic:**	**Detected at follow-up:**	**Detected in interval:**	**p. value:**
***Gender;***			.413
Female	8 (21.6%)	29 (78.4%)	
Male	15 (26.3%)′	42 (73.7%)	
***Deprivation score;***			.341
1(most deprived)	3 (60.0%)	2 (40.0%)	
2	3 (33.3%)	6 (66.7%)	
3	6 (27.3%)	16 (72.7%)	
4	7 (25.9%)	20 (74.15)	
5(least deprived)	4 (12.9%)	27 (87.1%)	
***Rurality;***			.571
Urban	14 (32.6%)	29 (67.4%)	
Rural	9 (17.6%)	42 (82.4%)	
***Melanoma type;***			.964
Superficial spreading	7 (25.9%)	20 (74.1%)	
Nodular malignant	8 (26.7%)	22 (73.3%)	
Lentigo maligna	1 (20.0%)	4 (80.0%)	
Acral lentiginous	1 (20.0%	4 (80.0%)	
Other	6 (22.2%)	21 (77.8%)	
***Breslow thickness;***			.252
4 mm or less	11 (20.0%)	44 (80.0%)	
>4 mm	12 (30.8%)	27 (69.2%)	
***Age at Dx;***			.142
Up to 65 years	12 (25.0%)	36 (75%)	
65 years and above	18 (39.1%)	28 (60.9%)	
***Time to recurrence;***			.008
12 months or less	14 (51.9%)	13 (48.1%)	
Beyond 12 months	16 (23.9%)	51 (76.2%)	

### Logistic regression

A binary logistic regression of mode of recurrence detection (at follow-up versus interval) was conducted (Table 4). Those who had a recurrence within one year were 3.433 (95% CIs 1.340-8.796) times more likely to be detected at structured follow up than in the interval. Following adjustment for other potential explanatory variables time to recurrence remained the only potentially explanatory variable significantly associated with mode of recurrence detection (OR2.891 (95% CIs 1.082-7.720).

**Table 4:** Binary logistic regression; odds ratio of having recurrence detected at routine follow-up versus interval, based on key variables and individual factors

	**Odds ratio:**	**95% CI for odds ratio:**	
		**Lower**	**Upper**
**Time to recurrence *(< 1 year)***	**3.433**	**1.340**	**8.796**
Gender *(male)*	1.295	.486	3.449
Age *(>65 years)*	1.351	.518	3.523
Melanoma type			
*(Nodular vs. superficial)*	.842	.274	2.586
*(Lentigo vs. superficial)*	.632	.088	4.532
*(Acral vs. superficial)*	1.684	.162	17.516
*(Other vs. superficial)*	.842	.267	2.660
Breslow thickness *(Thicker (>4 mm))*	1.667	.694	4.004
Rurality *(Urban)*	1.286	.539	3.067
Deprivation *(Deprived)*	1.750	.548	5.591
**Adjusted binary logistic regression:**			
**Time to recurrence**	**2.891**	**1.082**	**7.720**
Age	1.714	.666	4.415
Breslow thickness	1.443	.573	3.635
Rurality	1.377	.527	3.596
Deprivation	1.626	.454	5.816

Adjusted Binary Logistic Regression; adjusted for variables we deemed could influence our values.

## Discussion

### Summary of key findings

Approximately one-third of recurrent melanomas in this small sample were detected at a routine scheduled follow-up appointment. Of those presenting in the interval, the majority were detected by the patient themselves. Of potential predictors of mode of melanoma recurrence only time to recurrence was statistically significant, with people being much less likely to detect their own recurrence within the first year since diagnosis.

### Context with other literature

In this sample nearly one third of melanoma recurrences were detected at scheduled follow-up appointments. This accords with previous findings that 26-45% of melanoma recurrences are found at scheduled follow-up by a clinician [8,9,13]. Of the remaining two thirds the majority were detected by the patients themselves in the interval between follow-up visits. Again, this accords with previous findings which report that between 47-68% of recurrent melanomas are self-detected [8,9]. That almost two thirds of people in this sample, and similar proportions in previous studies, have detected their own recurrence supports the view expressed by some that the resources devoted to melanoma follow-up should be used to educate patients in the practice of TSSE, rather than being spent on blood tests and clinical imaging [8-10,12,23,24]. The 16 patients with emergency or incidental presentation of their melanoma might all represent missed opportunities to self-detect recurrent melanoma with effective TSSE. A digital method to prompting and support TSSE appears timely and warranted [13,14]. Implementing novel technological approaches to cancer can be costly [25]. Our data suggest this cost could be mitigated by targeting those most likely to benefit.

Previous researchers have established that adjusting to a diagnosis of cancer takes time [26]. It is striking that those within a year of diagnosis were less likely to detect their own recurrent melanoma. This finding fits well with the notion of taking time to adjust to melanoma and the practice of TSSE [27]. Perhaps those in the immediate post-diagnostic period have most to gain from intensive support with respect to TSSE. In this context it is reassuring to note that previous interventions to promote TSSE, demonstrate it can be increased by well designed educational interventions [17-20].

### Strengths and limitations

Our study contributes knowledge to an under-researched area, the epidemiology of recurrent melanoma. Our study was rigorous and based on high quality clinical databases and medical records. This was not an attempt to fully describe the epidemiology of recurrent melanoma in Northeast Scotland. Instead the authors were able to conduct a pragmatic study which provided invaluable information to inform the further development of a digital intervention to support TSSE in those diagnosed with cutaneous melanoma. The results are broadly in keeping with what has been published on melanoma recurrence before, but the authors were able to identify where such an intervention should be targeted to achieve maximum benefit in a way that previous research has not done.

This sample of patients was small from a relatively small area. The results may not be generalizable. However, two facts mitigate against this limitation. Firstly, in population terms melanoma recurrence is relatively rare and consequently its epidemiology is under-researched. Small studies such as this can signpost the way toward much needed larger studies. Secondly, proportions of follow-up versus self detected melanoma observed here are similar to those previously reported from other regions. This suggests that there is scope for others to add to our findings using similar small samples. A further related limitation is that the sample is dominated by men. Since men are arguably less likely to sustain TSSE this should be borne in mind as a potential confounder for which we have not been adequately able to control. As with all retrospective studies based upon medical records this study was limited by the availability and quality of records. This limitation is, however, common to almost all previous work on the epidemiology of recurrent melanoma, further highlighting the need for larger studies on the epidemiology of melanoma recurrence in future.

## Conclusions

Scheduled follow-up is important and effective in detecting recurrent melanoma particularly in the immediate post diagnostic period. Patients who are within one year of being diagnosed with a primary cutaneous melanoma are significantly less likely to detect their own recurrence, potentially placing them at risk of poorer outcomes. A digital intervention to prompt and support TSSE for people diagnosed with cutaneous melanoma should start immediately a patient enters follow-up. Such an intervention should not be viewed as an alternative to current structured follow-up and may become less necessary over time. An immediate research priority is to develop an effective and user friendly digital application to prompt and support TSSE in those newly diagnosed with cutaneous melanoma. Subsequent research should include a randomised trial to ensure that such an intervention can sustain TSSE in the longer term. Further large-scale research on the epidemiology of recurrent melanoma is also required.

## Competing interests

All authors declare that they have no competing interests to declare.

## Authors’ contributions

RA, PW, MN, and PM designed the study. RA and PW collected and managed the data. RA, PW and PM conducted data analysis. RA, PW, SH, MN, PM wrote the manuscript. PM is the guarantor. All authors read and approved the final manuscript.

## Pre-publication history

The pre-publication history for this paper can be accessed here:

http://www.biomedcentral.com/1471-5945/14/4/prepub
